# *Klebsiella* invasive liver abscess syndrome presenting with a central nervous system manifestation secondary to latent cholecystitis: a case report

**DOI:** 10.1186/s13256-022-03325-7

**Published:** 2022-06-07

**Authors:** Osamu Kinoshita, Takanari Okamoto, Takayuki Ota, Shun Takayama, Yuta Oi, Sachie Tanaka, Ichita Taniyama, Kei Naito, Yasuo Inoue

**Affiliations:** 1 Department of Surgery, Maizuru Medical Center, Aza Yukinaga 2410, Maizuru, Kyoto 6258502 Japan; 2 Department of Neurosurgery, Maizuru Medical Center, Aza Yukinaga 2410, Maizuru, Kyoto 6258502 Japan; 3 Department of Gastroenterology, Maizuru Medical Center, Aza Yukinaga 2410, Maizuru, Kyoto 6258502 Japan; 4 Department of Digestive surgery, Iseikai Hospital, Higashiyodogawa, Sugawara 6-2-25, Osaka, 5330022 Japan

**Keywords:** *Klebsiella*, Invasive liver abscess, Brain abscess, Cholecystitis

## Abstract

**Background:**

Brain abscess is a life-threatening event. Moreover, when *Klebsiella pneumoniae* is the cause, rapid diagnosis and appropriate treatment are required. *Klebsiella* invasive liver abscess syndrome, a bloodstream metastatic infection of potentially aggressive nature, has been recognized to cause infection in the central nervous system, and concern for *Klebsiella* liver abscess syndrome is increasing globally.

**Case presentation:**

A 73-year-old Japanese woman was admitted to the institution complaining of aggravated dysarthria and weakness in the right upper extremities with onset 5 days earlier. Magnetic resonance imaging revealed a brain abscess in the left basal ganglia, and abdominal computed tomography revealed a liver abscess in liver segment 7. The patient’s dysarthria symptoms became increasingly worse over the next few days, so surgical drainage via frontotemporal craniotomy was performed on admission day 3, and subsequent culture from the brain abscess showed growth of *Klebsiella pneumoniae*. On admission day 9, percutaneous transhepatic drainage of the liver segment 7 abscess was undertaken. The pus culture also showed growth of *Klebsiella pneumoniae*, thus associating the liver abscess with the brain abscess. Following long-term conservative treatment with antibiotics and abscess drainage, the liver abscess disappeared. However, the patient continuously presented with right upper quadrant pain, and abdominal computed tomography showed swelling of the gallbladder. Consequently, percutaneous transhepatic gallbladder drainage was initially administered, and the bile culture was also positive for *Klebsiella pneumoniae*. For radical treatment, a laparoscopic cholecystectomy was performed on admission day 99. The postoperative period was complicated by an intraabdominal abscess; however, conservative therapy was successful. She was subsequently discharged, and 12-month follow-up revealed no further sequelae.

**Conclusions:**

We describe a rare case of *Klebsiella* liver abscess syndrome, which first presented with a central nervous system manifestation. Our patient was successfully treated via an early surgical intervention and subsequent antibiotic therapy. Although surgical drainage remains the cornerstone treatment for brain abscess, when a brain abscess is found, and there is a high index of suspicion for the existence of a liver abscess, *Klebsiella* liver abscess syndrome should be considered as a possible diagnosis.

## Background

Brain abscess is a focal parenchymal infection of the brain [1]. It occurs in approximately 2% of intracranial masses in Western countries [2]. Although brain abscess itself is a sufficiently lethal event, when *Klebsiella pneumoniae* is the cause, rapid diagnosis and appropriate treatment are warranted to prevent metastatic complications [3, 4]. *Klebsiella* invasive liver abscess syndrome (KLA), which is characterized by liver abscesses and associated with metastatic complications, is reported to cause infection in the central nervous system, and concern for KLA is increasing globally. Although there have been few reports from Japan describing KLA, here we report an interesting case of KLA in a Japanese patient presenting with a central nervous system manifestation secondary to latent cholecystitis.

## Case presentation

**Fig. 1:**
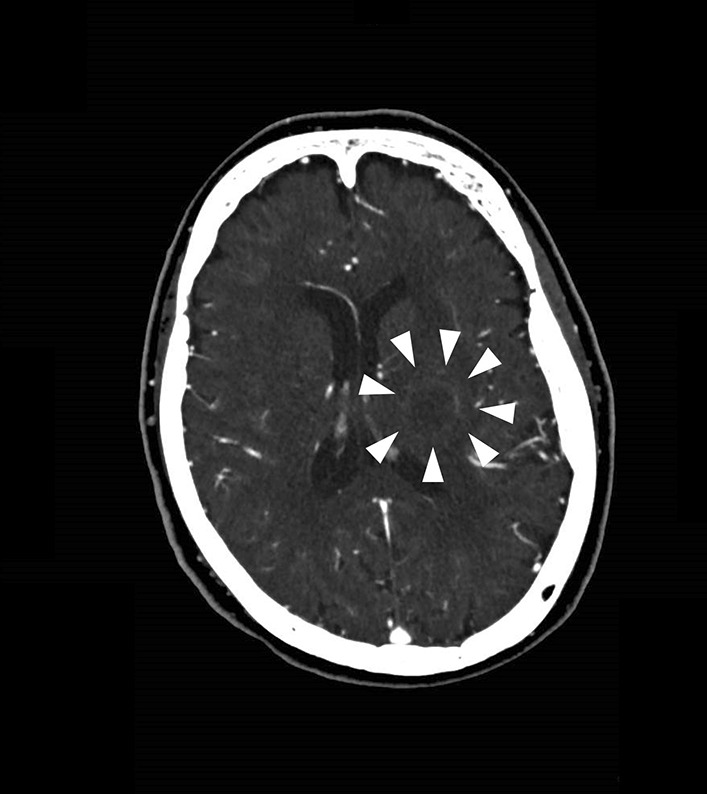
Initial computed tomography of the brain shows low-dense rounded walled lesion measuring 20×16 mm in the left basal ganglia (arrow head)

**Fig. 2:**
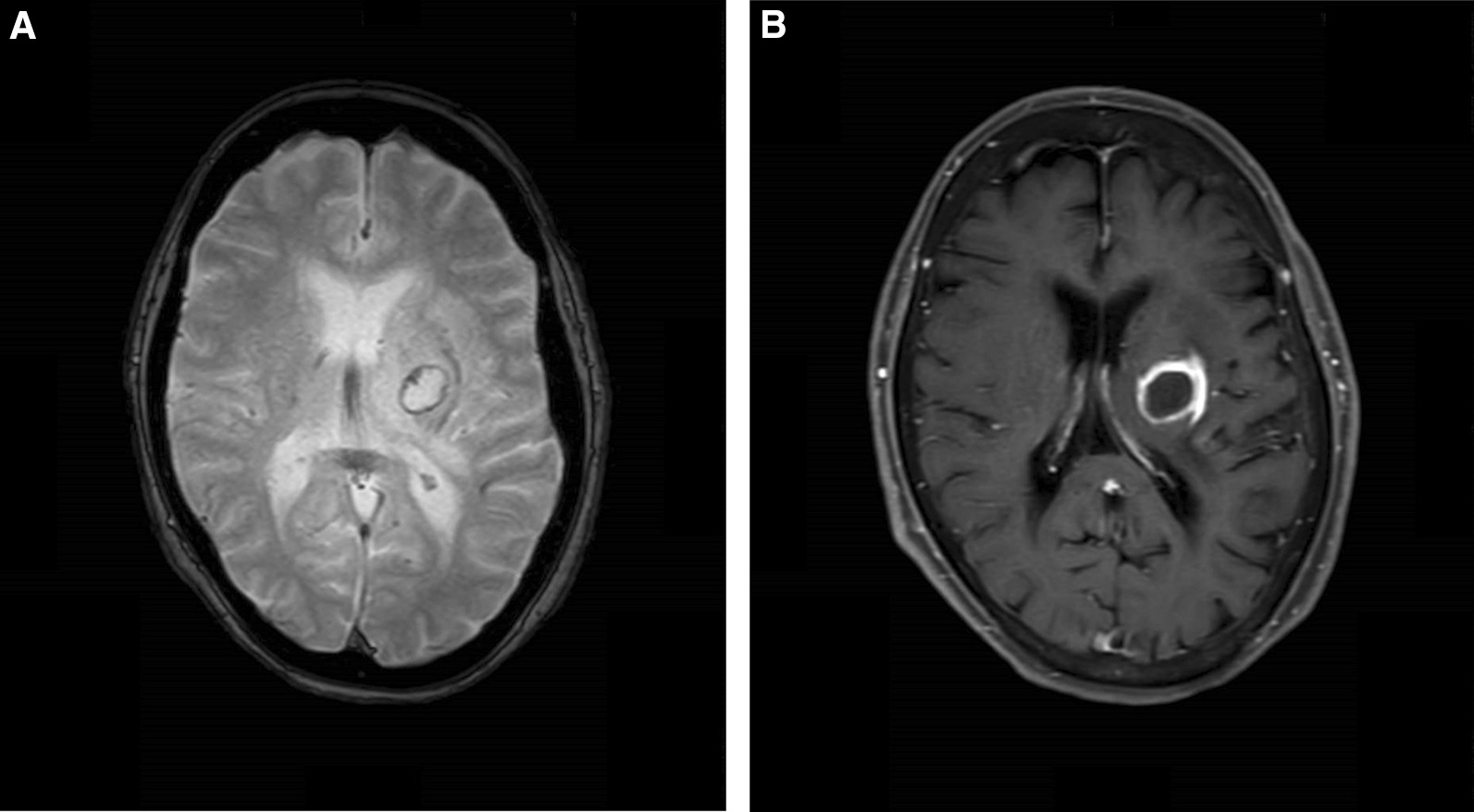
T2-weighted image shows rounded lesion with hypo-intense signal in the peripheral and hyper-intense signal in the center (left), and T1-weighted Gd-contrast enhancement shows well ring-enhanced cystic lesion (right)

**Fig. 3:**
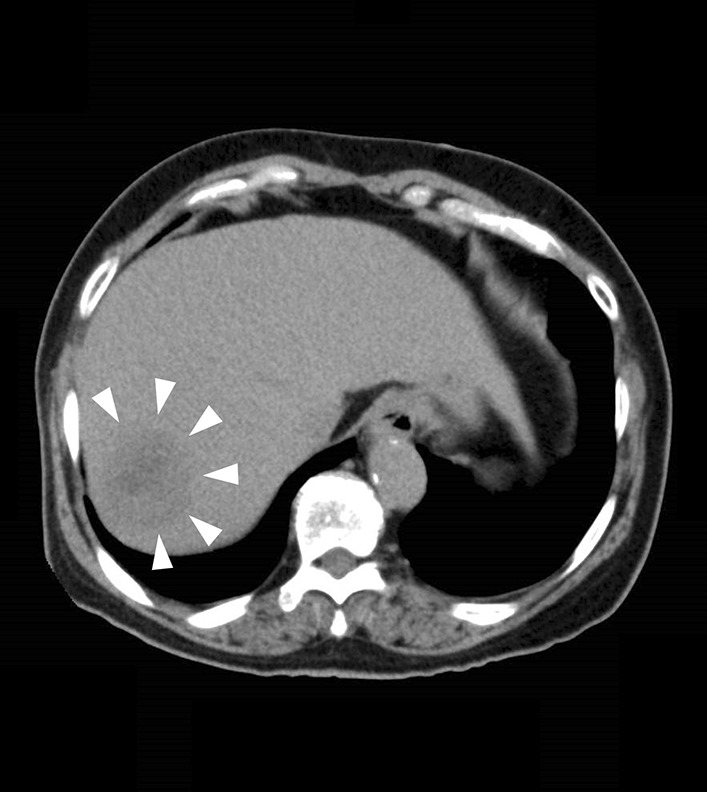
Plain computed tomography of the abdomen shows low density space-occupying lesion with largest measuring 50×37 mm (arrow head)

A 73-year-old Japanese woman with an unremarkable medical history and no previous diagnosis of opportunistic infection, was admitted to our institution complaining of aggravated dysarthria and weakness in the right upper extremities with onset 5 days earlier. Her general status was stable, and her vital signs were as follows: clear consciousness (Glasgow Coma Scale E4 V5 M6), body temperature 36.9 °C, blood pressure 128/92 mmHg, and pulse rate 89 beats per minute. On neurological examination, she had mild-to-moderate dysarthria and facial palsy. No other abnormal signs of cranial nerve involvement were evident, and her motor and sensory systems were intact with a manual muscle strength scale grade of 5/5 for the upper extremities. Her preliminary laboratory works were as follows: leukocytes 11,100/μl (percentage neutrophils 82.4%), C-reactive protein 3.48 mg/dl (normal range < 0.03 mg/dl), creatinine 0.89 mg/dl, and HbA1c (National Glycohemoglobin Standardization Program) 6.5%. Liver enzymes were in the reference range. Computed tomography (CT) of the brain showed a space-occupying lesion in the left basal ganglia (Fig. 1). Magnetic resonance imaging (MRI) revealed the lesion to be a brain abscess (Fig. 2a and b). Furthermore, a routine workup for the abdominal CT revealed a low-density space-occupying lesion in the liver (segment 7, S7), which strongly suggested a liver abscess (Fig. 3).

These results indicated simultaneous abscesses of the brain and liver. On admission, cultures of both spinal fluid and blood yielded no microorganism growth, and empirical treatment with 1 g meropenem three times a day plus 700 mg vancomycin three times a day was initiated. Nevertheless, the patient’s dysarthria symptoms became increasingly worse over the next few days, so surgical drainage via a frontotemporal craniotomy was performed on admission day 3. During the craniotomy, the left putaminal lesion was found to be filled with pus, and the subsequent culture showed the growth of multidrug-resistant *Klebsiella pneumoniae*, confirming a diagnosis of pyogenic brain abscess. The operation lasted 347 minutes, and intraoperative bleeding was 165 cm^3^. After surgery, the patient had slight residual neurological sequelae, including mild motor aphasia and weakness in the upper extremities.

On admission day 9, percutaneous transhepatic drainage of the liver (S7) abscess was undertaken. The pus culture also showed growth of multidrug-resistant *Klebsiella pneumoniae*, thus associating the liver abscess with the brain abscess. Following long-term conservative treatment with antibiotics and abscess drainage, the liver abscess disappeared.

**Fig. 4:**
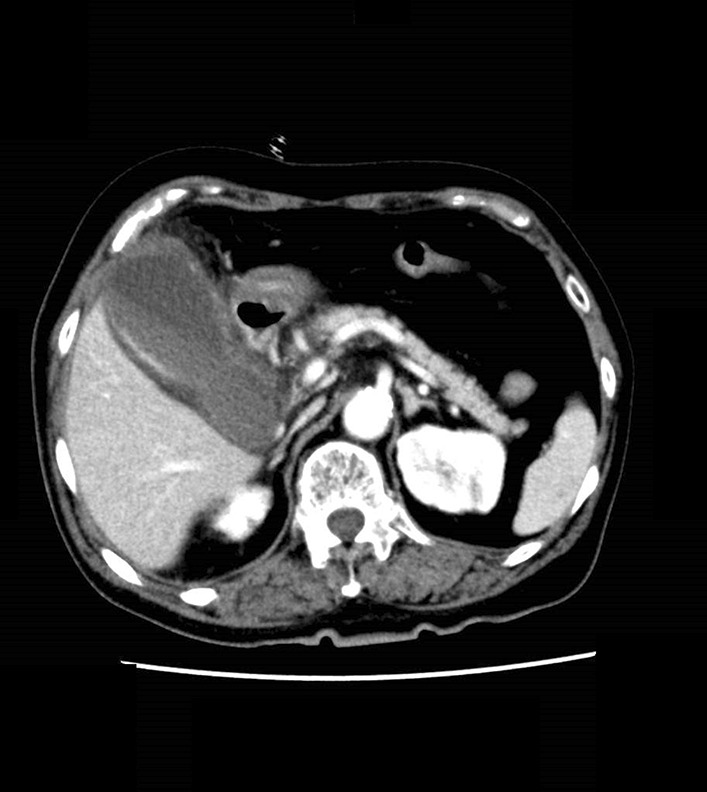
Contrast enhancement computed tomography

Although she received rehabilitative care after the brain surgery, the patient presented with right upper quadrant pain, nausea with vomiting, and low-grade fever during continuous hospitalization on admission day 74. Abdominal CT showed swelling of the gallbladder (Fig. 4), consistent with a diagnosis of acute phase on chronic cholecystitis. Conservative therapy with 1 g meropenem twice daily and percutaneous transhepatic gallbladder drainage (PTGBD) was initially administered, and the bile culture was also positive for multidrug-resistant *Klebsiella pneumoniae*. For radical treatment, the patient subsequently opted to undergo a cholecystectomy on admission day 99. A laparoscopic cholecystectomy was initially attempted; however, due to severe inflammatory adhesions and to mitigate excessive risk, a laparoscopic subtotal cholecystectomy was performed. The operation lasted 87 minutes, and there was little bleeding. A subsequent histopathological examination revealed developing granulation, residual inflammatory cells, and abscess formation in the thickened wall of the gallbladder, confirming a diagnosis of acalculous cholecystitis. Unfortunately, the postoperative period was complicated by an intraabdominal abscess. The causative bacterium of the intraabdominal abscess was multidrug-resistant *Enterococcus faecalis*, which was not consistent with the bacterium in the brain and liver abscesses, and it was treated with 700 mg vancomycin once daily. The patient was subsequently discharged, and 12-month follow-up revealed no further sequelae.

## Discussion

Brain abscess is a life-threatening event. Although the prevalence of brain abscess in Japan is not clear, the annual incidence of patients per 100,000 population is estimated to be 1.3 in Western countries [5]. Despite the development of antibiotic treatment, the overall death rate is approximately 10–20%, and many survivors have sequelae of convulsions, intelligence disorders, or local neurologic symptoms [2, 6]. In terms of brain abscess infection routes, the direct spread of a contiguous cervicofacial infection (for example, paranasal sinus, middle ear suppurative disease, decayed tooth) reportedly accounts for 50–80% of cases. Polymicrobial infection is thus common, and predominantly anaerobes, streptococci, Enterobacteriaceae, *Staphylococcus aureus*, and fungi are isolated. *Klebsiella pneumoniae* is rarely isolated (2%) [6].

*Klebsiella* liver abscess with developing bloodstream metastatic infection of a potentially aggressive nature was recognized as KLA, and was highlighted in the 1990s, raising concern among clinicians globally. KLA was first described in Taiwan in the 1980s [7]. Subsequently, a substantial number of cases were identified in East Asian countries, and evidence has since been accumulating. Although there has been lively discussion on KLA in conference proceedings in Japan, there is scant literature in Japanese on the condition. Our search using the keywords “Klebsiella,” “invasive liver abscess,” and “brain abscess/meningitis/endophthalmitis” for the period 1980–2020 identified only three Japanese-language case reports. KLA is currently considered an endemic disease in Taiwan [8, 9]; however, the response to future global transmission is an urgent priority, with concerns that it may emerge in Japan.

Siu *et al.* [4] reviewed aspects of KLA trends. Their clinical definition following substantial consensus was “*Klebsiella pneumonia* liver abscess with extrahepatic complications, especially central nerve system involvement, necrotizing fasciitis, or endophthalmitis.” In line with their definition, the abscess in the present report was identified as KLA. However, a limitation of the diagnosis in the present case was that a microbiological investigation was not undertaken, so the presence of the K1 or K2 serotype was not established. Interestingly, previous reports [4] have suggested that cases fulfilling both the clinical and microbiological definitions are strongly associated with a poor prognosis and warrant intensive treatment.

In the present case, acute cholecystitis was found at the end of the clinical course. Since the bile culture obtained from the PTGBD showed growth of *Klebsiella pneumoniae*, we presumed that the latent cholecystitis was associated with KLA. In our opinion, the subsequent cholecystectomy was reasonable for infection control; however, our search of the literature yielded no studies with a focus on the association between KLA and cholecystitis, so its significance remains unclear. Further studies are warranted.

Similar to the present report, a case report recently described a patient who initially presented with mild confusion and facial asymmetry and developed acalculous cholecystitis complicated by liver abscess and kidney injury several weeks later [10]. The authors diagnosed cholesterol embolization syndrome (CES), which is caused by atherosclerotic plaques that result in ischemic and inflammatory insults. While the case obviously differs from ours, the various clinical presentations and imaging of CES mimic KLA sufficiently to warrant close attention.

## Conclusions

In this report, we described a rare case of KLA, which first presented with central nervous system involvement in the form of aggravated dysarthria and weakness in the right upper extremities. Our patient was successfully treated via an early surgical intervention and subsequent antibiotic therapy. Although surgical drainage remains the cornerstone treatment for brain abscess, when a brain abscess is found, if there is a high index of suspicion for the existence of a liver abscess, KLA should be considered as a possible diagnosis.

## Data Availability

Nothing to declare in this article.

## Abbreviations

KLA: *Klebsiella* liver abscess syndrome
PTGBD: Percutaneous transhepatic gallbladder drainage
CT: Computed tomography
MRI: Magnetic resonance image
